# Epididymo-Orchitis Caused by POM-1 Metallo-β-Lactamase-Producing *Pseudomonas otitidis* in an Immunocompetent Patient: Case Report and Molecular Characterization

**DOI:** 10.3390/pathogens11121475

**Published:** 2022-12-05

**Authors:** Moayad Alqurashi, Abdulrahman Alsaedy, Bassam Alalwan, Maha Alzayer, Abdulrahman Alswaji, Liliane Okdah, Michel Doumith, Hosam Zowawi, Sameera Aljohani, Majed Alghoribi

**Affiliations:** 1 Department of Medicine, King Abdulaziz Medical City, Ministry of the National Guard-Health Affairs, Riyadh 11426, Saudi Arabia; 2 Division of Adult Infectious Diseases, Department of Medicine, King Abdulaziz Medical City, Ministry of the National Guard-Health Affairs, Riyadh 11426, Saudi Arabia; 3 Infectious Disease Research Department, King Abdullah International Medical Research Center, Riyadh 11481, Saudi Arabia; 4 College of Medicine, King Saud Bin Abdulaziz University of Health Sciences, Riyadh 11481, Saudi Arabia; 5 Department of Pathology and laboratory medicine, King Abdulaziz Medical City, Ministry of the National Guard-Health Affairs, Riyadh 11426, Saudi Arabia; 6 UQ Centre for Clinical Research, Faculty of Medicine, University of Queensland, Brisbane, QLD 4029, Australia

**Keywords:** *Pseudomonas otitidis*, POM-1 Metallo-β-lactamase MBL-1, epididymo-orchitis, whole genome sequencing, Saudi Arabia

## Abstract

*Pseudomonas otitidis* is a rare and unique species among the *Pseudomonas* genus that has not been previously reported as a cause of male genitourinary tract infection. In this report, we describe a case of a 20-year-old immunocompetent male who presented with recurrent epididymo-orchitis, which was initially misidentified as Vibrio vulnificus and treated successfully. The causative agent could not be identified appropriately using the available routine methods, but a final identification was established using 16S rRNA targeted sequencing followed by whole-genome sequencing.

## 1. Introduction

Epididymo-orchitis is the most common cause of acute scrotum in young men [1]. Sexually active men are more prone to infections with *Chlamydia trachomatis* and *Neisseria gonorrhoeae*, while children and the elderly are usually prone to coliform bacteria such as *Escherichia coli* [1]. The infection of the testes by *Pseudomonas aeruginosa* is rare and has been estimated to be between 5% and 14% [2]. *Pseudomonas otitidis*, a rare species of the *Pseudomonas* genus, has not been previously reported as a cause of male genitourinary tract infection. However, little is known about the clinical importance, diagnostics, antimicrobial susceptibility, and genomic characterization of *P. otitidis* in clinical settings. Here, we discuss a difficult-to-identify *P. otitidis* that presented as acute epididymo-orchitis with molecular characterizations of the isolate and in the literature review.

## 2. Case Report

A 20-year-old previously healthy single man presented with fever and a tender right testicular swelling for two days. Three weeks prior to this presentation, he had an episode of hematuria with dysuria that was treated with an over-the-counter antibiotic as a presumed urinary tract infection (UTI). He was vitally stable, with unremarkable physical examination other than a tender, swollen right testicle without obvious wounds or discharging sinuses. The white blood cell (WBC) count was elevated at 22 × 10^9^/L (neutrophils 89%), and the erythrocyte sedimentation rate was 73 mm/h. Urine analysis and subsequent culture were negative for UTI, and gonococcal screening took place using the nuclear amplification technique. A testicular ultrasound demonstrated an epididymo-orchitis with abscess formation, for which he was treated conservatively. He received nine days of meropenem 1000 mg intravenously (IV) every eight hours. As his symptoms improved and the testicular swelling decreased, he was discharged home on oral ciprofloxacin 500 mg twice per day to complete a ten-day course.

However, the patient was re-presented to the emergency room seven days post-discharge with increasing non-tender swelling of the same testicle. Ultrasound confirmed the recurrence of the abscess. The WBC count in this presentation was normal at 7.6 × 10^9^/L. Although urine analysis was not suggestive of UTI and, subsequently, the culture was negative, meropenem 1000 mg IV every eight hours was restarted empirically, and surgical drainage of the abscess was performed. The pus drained after the incision of the tunica albuginea (the deepest layer of the testes), and tissue taken from the seminiferous tubules was sent for culture. The laboratory initially misidentified the organism as *Vibrio vulnificus* using VITEK-MS (bioMérieux, La Balme Les Grottes, France) but only with a very low confidence of 35%. Phenotypically, the isolate was generally susceptible to most tested antibiotics (Table 1).

A review of the risk factors for developing such an infection excluded water-sport exposure, unusual sexual activities, or shellfish consumption. Workups for hepatitis B and C, HIV, and liver diseases were negative. However, he had a history of Tilapia fish consumption shortly before the initial onset of the patient’s symptoms. This type of fish has a particular association with *V. vulnificus* biotype 3 infections [4]. Hence, the patient was discharged home on oral doxycycline 100 mg twice daily for six weeks. On follow-up four months later, the patient suffered a recurrence in the form of swelling and testicular pain that was treated successfully with another course of doxycycline monotherapy for another six weeks. The patient was healthy and has not suffered any further recurrences since the attack in 2016.

Interestingly, returning to the saved bacterial sample in the laboratory to determine the *V. vulnificus* biotype was surprisingly difficult. Repeated attempts to re-identify the organism using standard laboratory techniques (API20E, API20NE, VITEK-2 GN, and VITEK-MS) provided conflicting results. On some occasions, the organism was not identified, whilst in others, different identities were detected. The oxidase test was positive. Microscopic examination revealed a gram-negative rod (not curved as expected of *Vibrio* spp.) which was seen as individual cells.

For further molecular identification, the isolate was sent to the infectious diseases research department at the King Abdullah International Medical Research Center (KAIMRC). The isolate was cultured on nutrient agar at 37 °C for 24 h, and a pure single colony was used for DNA extraction according to the manufacturer’s instructions using the MagNA Pure compact nucleic acid isolation kit I (Roche, Switzerland).

Molecular identification using the 16S rRNA targeted sequencing was performed. The full length of the 16S rRNA gene was amplified using two conservative primers (27F 5′-AGAGTTTGATCCTGGCTCAG-3′) and (1492R 5′-GGTTACCTTGTTACGACTT-3′) [5,6]. The amplified amplicon was then subjected to Sanger sequencing, and sequence analyses were performed using QIAGEN CLC Main Workbench (version 20.0.4). The consensus sequence was aligned against ribosomal RNA gene databases to identify the bacterial taxonomy, including SILVA- SINA (version 1.2.11) [7] and EzBioCloud databases (version 2021.07.07) [8]. The 16S sequence was identified as *Pseudomonas otitidis* with 98% similarity.

In addition, whole-genome sequencing (WGS) was performed on the MiSeq Illumina instrument using the Nextera XT DNA library preparation kit (Illumina, USA) following the 2 × 300 paired-end protocols. The generated sequencing data were assembled using SPAdes software (version 3.15.3) [9]. The generated contigs were constructed using Mauve Contig Mover (version 2.4.0) to order the draft genome’s contigs to complete the reference genome of *P. otitidis* MrB4 isolates (GenBank accession numbers AP022642.1).

The total genome size of the isolate (hereafter referred to as POSA1) was 6,258,324 bp with a G+C content of 66.73%. The identification of antibiotic resistance genes was determined with ABRicate (https://github.com/tseemann/abricate (accessed on 1 November 2022)) (version 0.9.8) using the ResFinder (version 2.1) database [10]. The WGS resistome analysis showed that the isolate harbored only one resistance gene coding for *P. otitidis* Metallo-β-lactamase MBL-1 (named POM-1), conferring the resistance to carbapenems. The *bla*_POM-1_ gene is located in the contigs, which is a part of the bacterial chromosome. The genomic environment of the *bla*_POM-1_ gene shows no mobile genetic elements, such as insertion sequence or transposon, suggesting that *bla*_POM-1_ is an intrinsic gene of *P. otitidis*.

## 3. Discussion

For *Pseudomonas* spp. to cause epididymo-orchitis, it has to invade through the retrograde passage of fluids to the vas deferens, which is the most common cause of the infection, or through the bloodstream to a predisposed anatomical abnormality in the genitourinary tract [11,12]. Although pseudomonal infection of the testes is rare, it should be suspected in patients with a history of ano-receptive intercourse or patients with a poor to no response when using empirical antibiotics, where gram-negative coverage, including anti-pseudomonal, should be added [12]. The presence of an abscess is an indication of incision and drainage with possible orchiectomy in severe cases [13]. Evidence for the best antimicrobial regimen in the case of epididymo-orchitis is lacking, and the selection of appropriate antibiotics should be individualized to each case [14].

*Pseudomonas otitidis* was first discovered in 2002 by Roland P. and Stroman D. [15] and described in detail in 2006 by Clark L. et al. during a clinical study of patients with otic infections (otitis externa, otitis media, etc.) [16]. It is a gram-negative, motile rod typically positive for oxidase, and they have non-fluorescent pigment-producing colonies for most but not all strains [16,17]. It belongs to the family *Pseudomonadaceae* as part of the *P. aeruginosa* group (*P. aeruginosa*, *P. alcaligenes*, and *P. otitidis*) [18,19]. The closest *pseudomonal* spp. phenotypically and genotypically in the phylogenetic tree using the neighbor-joining method is *P. aeruginosa* (98.6% 16S rRNA gene sequence similarity) [16,20]. This organism can constitutively produce a novel subclass of the B3 Metallo-β-lactamase (MBL) enzyme, which is named *bla*_POM_ (after *P. otitidis* MBL) [20,21]. Unlike all other pathogenic *Pseudomonas* species, *P. otitidis* is the first example endowed with a resident MBL gene in the absence of other β-lactamase genes [20]. Some strains share more than 80% of the virulence factors of *P. aeruginosa*, including the ability to colonize, evade the immune system, and cause lesions and other human diseases [22].

The POM-1 protein is closely related to other known B3 MBLs, such as L1, with up to 64% similarity, suggesting a closer evolutionary ancestry, while having a lower similarity to other B3 MBLs, such as THIN-B, CAU-1, BJP-1, FEZ-1, GOB-1, and CAR-1 [20]. Phenotypically, this gene has variable expression levels of up to a five-fold variation in POM-1 protein production between different strains, independent of the β-lactam exposure level [20]. It can also lead to varying degrees of carbapenem resistance, especially imipenem and meropenem while being sensitive to piperacillin/tazobactam, ceftazidime, and aztreonam, depending on which minimum inhibitory concentration (MIC) cut-off is used (CLSI vs. EUCAST) [20]. It can also have a discrepant carbapenem profile in some strains, with reduced susceptibility to meropenem while being susceptible to imipenem [22]. Inoculum size was also found to affect carbapenem susceptibility, significantly raising the MICs of imipenem and meropenem by 16-fold and 64-fold, respectively, when the inoculum size was increased from 10^5^ to 10^7^ CFU, respectively [20]. The impact of POM-1 production on β-lactam resistance was investigated by subcloning a gene fragment that included the *bla*_POM-1_ gene into a shuttle plasmid used to transform two bacteria (*E. coli* and *P. aeruginosa*). It resulted in MBL production in both transformants but with different activities and effects on the MIC values compared to *P. otitidis* [20]. Some environmental strains were mercury resistant, which is one of the most hazardous metals in the environment [23].

*P. otitidis* was isolated from many environmental sources, such as soil, plants [17], food sources [24], different water bodies, sewage [25], and public toilets [18]. A recent article reported the presence of *P. otitidis* in the sewage water of Taif city in Saudi Arabia [26]. Basically, it can be isolated where environmental pollution is present [27]. Clinically, most isolates were reported from patients with otic infections [16,28], yet there were three published reports of non-otic infections with this organism. Two immunocompromised patients suffered necrotizing fasciitis and pan-peritonitis due to *P. otitidis,* which was initially misidentified as *P. aeruginosa* [29]. The third was an immunocompetent patient with *P. otitidis* bacteremia and pneumonia, which was detected using MALDI-TOF MS and confirmed using 16s rRNA sequencing [30]. This case report is the fourth case in the literature to be reported of a non-otic infection due to this organism, which raises doubt about the rarity of this species in clinical samples since routine methods are not usually able to detect it properly.

Discrepancies in organism identification by conventional and commercially available methods such as VITEK-2 and MALDI-TOF MS have been described and studied in the literature with disagreement; there was no identification or misidentification between the two methods by around 8.6% in one study, which was more pronounced in the gram-negative group [31]. Other studies have also confirmed similar observations when three biochemically based systems and two commercially MALDI-TOF MS systems were compared to the gold-standard test of WGS in identifying *Enterococcus* species; although VITEK-2 results were consistent with the VITEK-MS results, both systems misidentified *Enterococcus faecalis* by 87% [32]. Uncertainty in diagnosing *P. otitidis* specifically was reported despite the use of VITEK-MS [33]. This is due to the lack of identifying information in the current VITEK-MS database [34]. The Bruker MALDI-Biotyper (Bruker Daltonics, Bremen, Germany), on the other hand, can identify *P. otitidis*, as in the previously reported case reports [29,30].

## 4. Conclusions

To our knowledge, there are no prior reports in the literature of genital infection with *P. otitidis* in an immunocompetent patient. The misidentification of *P. otitidis* as *P. aeruginosa* or other species is relatively common since commercially available methods may not be able to identify it. In order to correctly identify *P. otitidis*, advanced molecular identification (16s rRNA sequencing, WGS, etc.) is warranted, especially if the isolate has high MIC or is resistant to carbapenems while being susceptible to piperacillin/tazobactam, ceftazidime, and aztreonam phenotypically.

## Figures and Tables

**Table 1:** Antimicrobial susceptibility.

Antibiotic	MIC (μg/mL)	Interpretation ^*^	Antibiotic	MIC (μg/mL)	Interpretation ^*^
Ampicillin/Sulbactam	≥32	Resistant	Amikacin	≤2	Susceptible
Piperacillin/Tazobactam	16	Susceptible	Gentamicin	≤1	Susceptible
Ceftazidime	4	Susceptible	Tobramycin	≤1	Susceptible
Cefepime	≤1	Susceptible	Ciprofloxacin	0.5	Susceptible
Imipenem	0.5	Susceptible	Levofloxacin	0.25	Susceptible
Meropenem	4	Resistant	Minocycline	4	Susceptible
Aztreonam	16	Intermediate	Trimethoprim/sulfamethoxazole	≤20	Susceptible

* MIC interpretation of several antibiotics using VITEK-2 with *Vibrio vulnificus* as a selected organism. Results were interpreted using CLSI breakpoints [3]. Abbreviations: MIC = minimum inhibitory concentration; CLSI = clinical and laboratory standards institute.

## Data Availability

Whole genome sequence data were deposited in NCBI under BioProject accession number PRJNA779563 and SRA number SRR16915391 (https://www.ncbi.nlm.nih.gov/bioproject/PRJNA779563 (accessed on 1 November 2022)).
